# Mechanical Stretch and PI3K Signaling Link Cell Migration and Proliferation to Coordinate Epithelial Tubule Morphogenesis in the Zebrafish Pronephros

**DOI:** 10.1371/journal.pone.0039992

**Published:** 2012-07-18

**Authors:** Aleksandr Vasilyev, Yan Liu, Nathan Hellman, Narendra Pathak, Iain A. Drummond

**Affiliations:** 1 Department of Pathology, Massachusetts General Hospital, Charlestown, Massachusetts, United States of America; 2 Nephrology Division, Massachusetts General Hospital, Charlestown, Massachusetts, United States of America; 3 Department of Genetics, Harvard Medical School, Boston, Massachusetts, United States of America; MRC, University College of London, United Kingdom

## Abstract

Organ development leads to the emergence of organ function, which in turn can impact developmental processes. Here we show that fluid flow-induced collective epithelial migration during kidney nephron morphogenesis induces cell stretch that in turn signals epithelial proliferation. Increased cell proliferation was dependent on PI3K signaling. Inhibiting epithelial proliferation by blocking PI3K or CDK4/Cyclin D1 activity arrested cell migration prematurely and caused a marked overstretching of the distal nephron tubule. Computational modeling of the involved cell processes predicted major morphological and kinetic outcomes observed experimentally under a variety of conditions. Overall, our findings suggest that kidney development is a recursive process where emerging organ function “feeds back” to the developmental program to influence fundamental cellular events such as cell migration and proliferation, thus defining final organ morphology.

## Introduction

It is well established that embryogenesis and cell specification can be controlled by developmental morphogens and sequential, tissue-specific changes in gene expression. It is equally clear that to achieve the higher order structure during organ morphogenesis, cell fate specification must be linked to cell rearrangement, migration, and other physical processes that determine the ultimate organ shape and function [1], [2]. Mechanical interactions have been shown to guide lung [3], heart and vasculature [4], [5], hematopoietic [6], [7] and musculoskeletal [8], [9] system development. At the same time, the cellular mechanical environment can be directly affected by the onset of organ function, which unfolds during organ morphogenesis. In the kidney, vascular shear force in capillaries is required for remodeling the glomerulus and formation of the glomerular capillary tuft that initiates blood filtration [10]. Subsequent fluid filtration and flow within tubules is essential for normal kidney development and impeding fluid flow by obstruction leads to kidney dysplasia [11]. We have previously shown that fluid shear force in the lumen of zebrafish kidney tubules is required for nephron morphogenesis as it initiates collective tubule cell migration that accounts for the convoluted shape of mature proximal tubules and the final position of nephron segment boundaries [12]. Here we have investigated how collective migration in the zebrafish pronephros is coupled to epithelial cell proliferation during nephron morphogenesis. Our results suggest that migration-induced cell stretch plays a key role in signaling cell proliferation to replace migrating kidney cells. The findings indicate that physical interactions between cells guide complex morphogenetic processes during kidney organogenesis and that final kidney form is ultimately governed by kidney function.

## Results

### Cell Proliferation Occurs in Distinct Domains of the Developing Nephron

**Figure 1:**
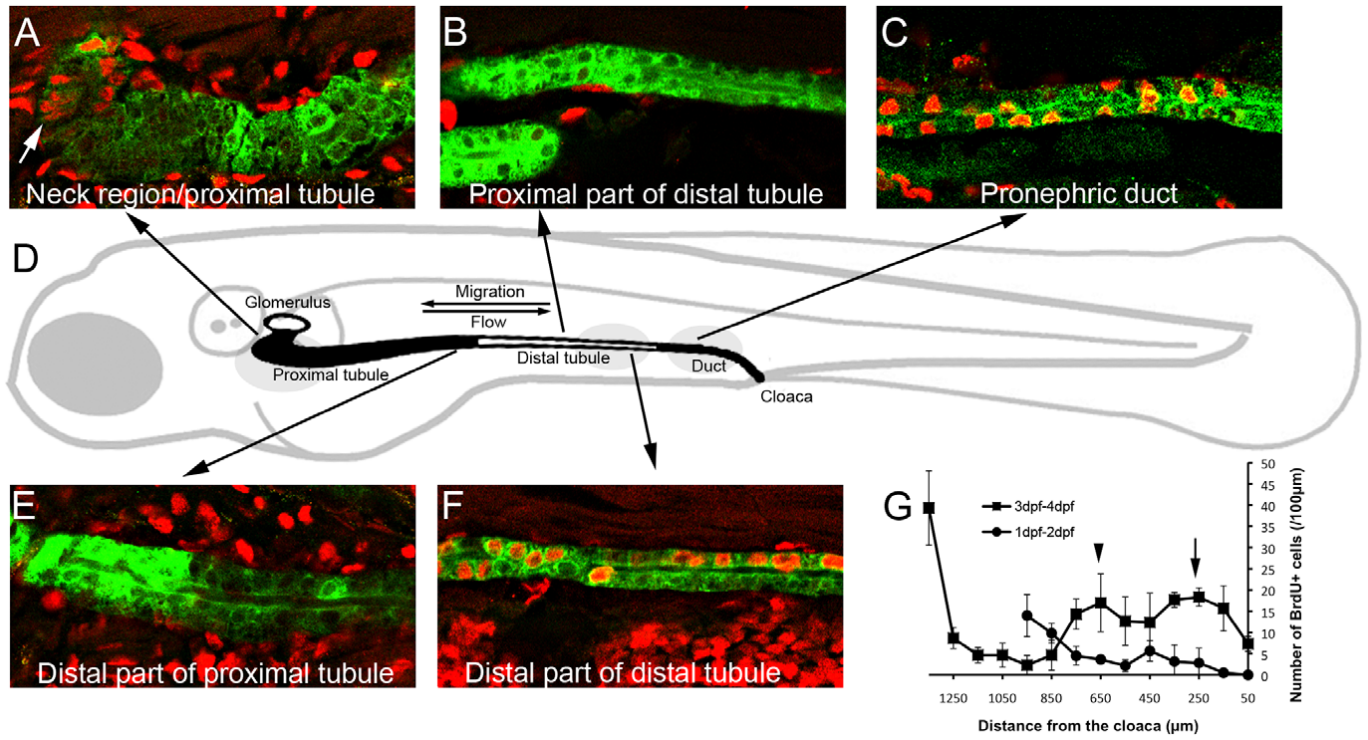
Pronephric epithelial proliferation. (A–C,E,F) Red: anti-BrdU staining, Green: anti-GFP staining. BrdU incorporation was measured for 24 h, between 48 and 72 hpf. (A) Proliferation in the proximal tubule is localized to the neck region (arrow). (B) Proliferation in the anterior part of the distal tubule is low. (C) Proliferation in the *ret1*-positive pronephric duct segment is high. (D) Cartoon depicting zebrafish pronephros. The proximal tubule (ET33d10 GFP positive domain) and the pronephric duct (*ret1*:GFP positive domain) are shaded black. The glomerulus and the distal tubule (ET11-9 GFP positive domain) are not shaded. (E) Proliferation in the posterior proximal tubule is low. (F) Proliferation in the posterior distal tubule is high. (G) Quantification of pronephric epithelial proliferation after 24 hour BrdU incorporation. The length of the tubule is plotted on the horizontal axis (measured from the cloaca). The linear density of BrdU positive nuclei (per 100 µm tubule length) is plotted on the vertical axis. Squares: pronephric proliferation between 3 and 4 dpf (n = 3). Circles: proliferation between 1 and 2 dpf (n = 3). See also Figure S1.

Previously we showed that kidney morphogenesis in the zebrafish is dependent on collective epithelial cell migration toward the proximal (anterior) pole of the nephron. The rate of migration is much higher in the proximal vs. the distal kidney [12], resulting in stretching of the distal kidney epithelium (movie S1). If left uncompensated, cell migration would be expected to lead to significant distortion of the distal kidney. A potential compensatory mechanism that would allow for lengthening of the distal nephron is cell proliferation. To test this hypothesis we first examined the rate of pronephric epithelial proliferation as a function of position within the nephron. Three distinct domains of cell proliferation were identified during the period of observation, between 1 and 5 dpf (Fig. 1). A proximal domain was observed in the segment adjacent to the glomerulus and was consistently present after 1 dpf, (Fig. 1 A, G). A second domain was located in the ret1 positive pronephric duct and was pronounced between 2 and 4 dpf (Fig. 1 C, G-arrow). The third domain of proliferation was observed in the distal tubule after 2 dpf (Fig. 1 F, G-arrowhead). This domain spatially correlated with the nephron segment exhibiting the greatest dynamic change in cell migration rate (from 2 µm/hr to >6 µm/hr, [12], movie S1). Since the migrating epithelial cells remain physically linked by adherens junctions, cells in the distal nephron are subjected to significant longitudinal stretch (defined as an increase in cell inter-nuclear distance in the absence of cell hypertrophy). Interestingly, the domain of cell proliferation in the distal tubule followed the actively migrating segment in the distal to proximal direction, shifting by approximately 100 µm per 24 h (Fig. S1).

### Cell Proliferation is Coupled to Cell Migration through Stretch and PI3K Signaling

**Figure 2:**
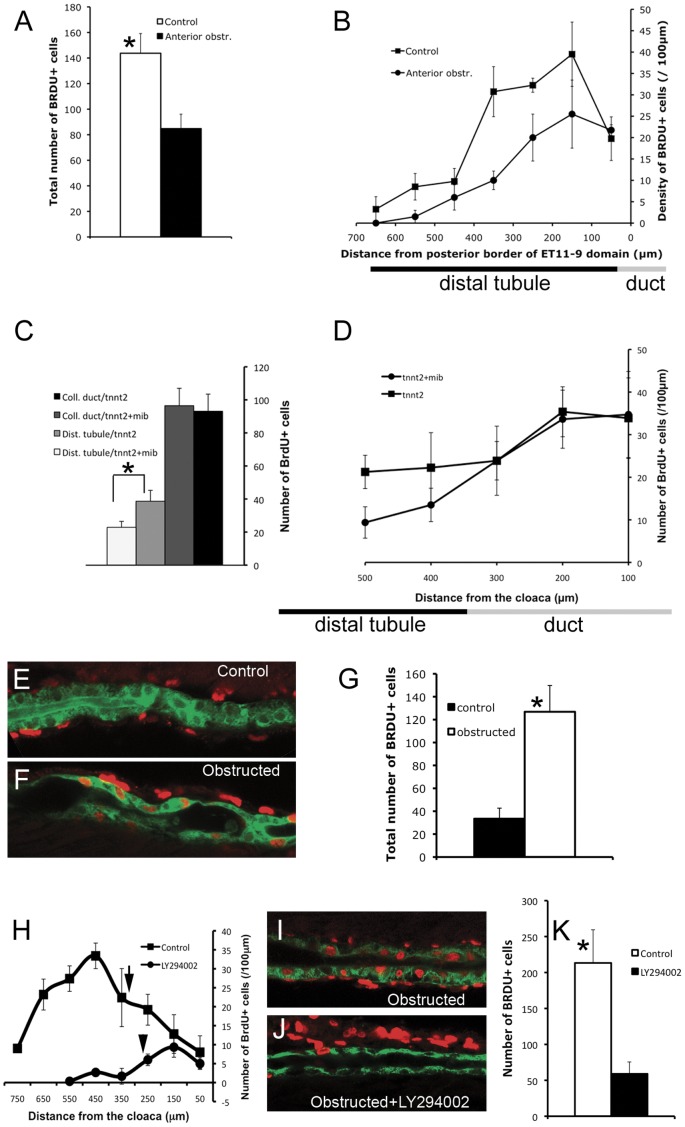
Collective epithelial migration stimulates cell proliferation in the distal tubule. (A, B) Cell proliferation in the distal tubule (ET11-9 GFP domain) after anterior obstruction. Obstruction was induced at 30 hpf, BrdU incorporation was assessed between 2 and 3 dpf. (A) Total number of BrdU+ nuclei in the distal ET11-9 domain. White bar: control (n = 4), black bar: anterior obstruction (n = 4). P = 0.01. (B) Spatial distribution of the BrdU-positive nuclei per 100 µm length of the distal tubule (measured from the posterior border of the ET11-9 domain). Squares: control (n = 4), Circles: anterior obstruction (n = 4). (C,D) Cell proliferation in the distal nephron in *mindbomb* mutants. *mindbomb* (*mib*) heterozygotes were in-crossed and injected with *tnnt2* morpholino to control for vascular defects in *mib* mutants. BrdU incorporation was assessed between 2 and 3 dpf. Homozygous *mib* mutants were separated from their siblings based on their axis curvature phenotype. (E) Total amount of BrdU incorporation in pronephric duct (black bar: control, n = 8; dark-grey bar: *mindbomb*, n = 8; P>0.05) and in the posterior distal tubule (light grey bar: control, n = 8; white bar: *mindbomb*, n = 8; P<0.01). (D) Linear density of the BrdU+ nuclei. Squares: *tnnt2MO* only control (n = 4); circles: *mindbomb* +*tnnt2MO* (n = 4). The underlying bars (B,D) indicate the position of distal tubule/pronephric duct border. (E) 24 hour BrdU incorporation in the posterior proximal tubule (2–3 dpf). (F) BrdU incorporation during 24 hour post-obstruction (2–3 dpf). (G) total number of BrdU positive nuclei in the distal 600 µm of proximal tubule. Black bar: control (n = 4), white bar: obstructed nephrons (n = 8); P<0.01. (H) Proliferation profile in control ET11-9/*Tg(atp1a1a.4:GFP)* transgenic fish compared to LY294002 treated fish (2–3 dpf). Arrows point to the location of distal tubule/pronephric duct interface. (I) Up-regulation of BrdU incorporation in stretched proximal tubule between 12 and 36 hours post-obstruction. (J) BrdU incorporation was markedly reduced in LY294002 treated, obstructed tubules. (K) Total number of BrdU-positive nuclei in the anterior 500 µm of the proximal tubule. White bar: BrdU incorporation in obstructed nephron/−LY294002 (n = 3), black bar: BrdU incorporation in obstructed nephron/+LY294002 (n = 3); P<0.05. (E,F) Green: GFP (ET33d10 GFP); (I,J) Green – cadherin17; (E,F,I,J) Red: BrdU.

Since mechanical stretch is known to be a stimulus for cell proliferation in other systems [13], we hypothesized that longitudinal epithelial stretch resulting from divergent rates of cell migration could stimulate cell proliferation in the distal tubule. To test the hypothesis, we stopped proximal migration by obstructing the kidney immediately distal to the proximal tubule. This manipulation blocks fluid flow and inhibits pronephric migration without causing tubular dilatation [12]. Anterior (proximal) nephron obstruction significantly reduced cell proliferation in the distal tubule (Fig. 2 A, B). However, the reduction in cell proliferation could be due to the absence of mitogenic factors that are normally delivered to the distal nephron by luminal fluid flow. To address this concern, we took a genetic approach and screened existing mutants for defective pronephric cell migration in an otherwise uninjured kidney. We found that the Notch pathway mutant *mindbomb* (*mib*) [14] lacked normal proximally directed pronephric epithelial migration (movie S2). *mindbomb* homozygotes exhibited significantly reduced cell proliferation in the distal tubule compared with wild-type siblings, further supporting the conclusion that distal tubule proliferation is stimulated by epithelial cell migration and resulting longitudinal stretch (Fig. 2 C, D). Both groups were injected with *tnnt2* morpholino to control for vascular defects in *mib* mutants. The absence of distal tubule proliferation in *mindbomb* mutants was not due to a cell autonomous defect caused by absence of Notch signaling since *mindbomb* tubules subjected to obstruction and associated cell stretch still proliferated robustly (Fig. S3 and See below). The results in Fig. 2 C, D also demonstrated that, in contrast to the distal tubule segment, cell proliferation in the duct segment occurred independently of pronephric migration. Similarly, the proximal domain of cell proliferation (next to the glomerulus) was insensitive to cell migration and luminal fluid flow. We used *tnnt2* morphants and compared them to control morpholino injected fish. Assessing proximal tubule proliferation showed no statistically significant difference between control fish and *tnnt2* morphants (Fig. S2 A, B). This result also supported our observation [12] that proximal tubule convolution is primarily driven by collective cell migration (and not cell proliferation): *tnnt2* morphants lack proximal component of cell migration [12] and fail to develop proximal convolution despite intact cell proliferation in the proximal segment.

Although pronephric migration starts at 29 hpf [12], we did not observe an increase in BrdU incorporation in distal tubule during the 24–48 hpf time window (Fig. 1 G), while the 48–72 hpf window showed very active distal tubule BrdU incorporation (Fig. 2 H). This suggested that proliferative response is not immediate, but that there is a delay of a few hours between an onset of cell migration and an onset of cell proliferation in distal tubule.

To test whether stretch can induce cell proliferation independently of cell migration we performed distal nephron obstruction in the ET33d10 transgenic that marks the proximal tubule with GFP expression [15], [16]. The proximal tubule outside of the peri-glomerular region does not actively proliferate during normal morphogenesis (Fig. 1 E). In obstructed nephrons, luminal expansion begins within minutes and results in stretching of tubule epithelial cells. BrdU incorporation studies revealed that obstruction-induced cell stretch alone was sufficient to induce a dramatic increase in cell proliferation in the proximal tubule (Fig. 2 E, F, G). These experiments also showed that at least 24 hr post-obstruction was needed to observe a significant proliferative response, further suggesting that the proliferative response is delayed with respect to cell stretch. At the same time, the amount of stretch induced by distal obstruction did not depend on cell proliferation (Fig. S4).

While cell stretch appears to be sufficient to signal proliferation, the signal transduction pathways linking cell migration, cell stretch and cell proliferation in nephron morphogenesis are not known. PI3K signaling is known to be activated in stretched regenerating wounded epithelia [17], [18]; it is also an important proliferative factor. Thus, we tested whether chemical inhibition of PI3K signaling would result in uncoupling of cell migration and cell proliferation. Our results showed that LY294002 treatment [19] resulted in marked reduction in cell proliferation specifically in stretched distal tubule cells (Fig. 2 H). Furthermore, while mechanical obstruction of the kidney resulted in significant up-regulation of cell proliferation in the stretched epithelium (Fig. 2 E–G), treating obstructed larvae with LY294002 significantly blunted this proliferative response but did not affect cell proliferation in neighboring mesenchymal cells, indicating that PI3K signaling was specifically associated with stretch-induced cell proliferation (Fig. 2 I–K). Overall these data support our conclusion that cell migration stimulates epithelial cell proliferation specifically in distal tubule and that cell stretch serves as a mechanical factor linking the two processes, dependent on intact Pi3K signaling.

### Cell Proliferation is Required for Nephron Morphogenesis

**Figure 3:**
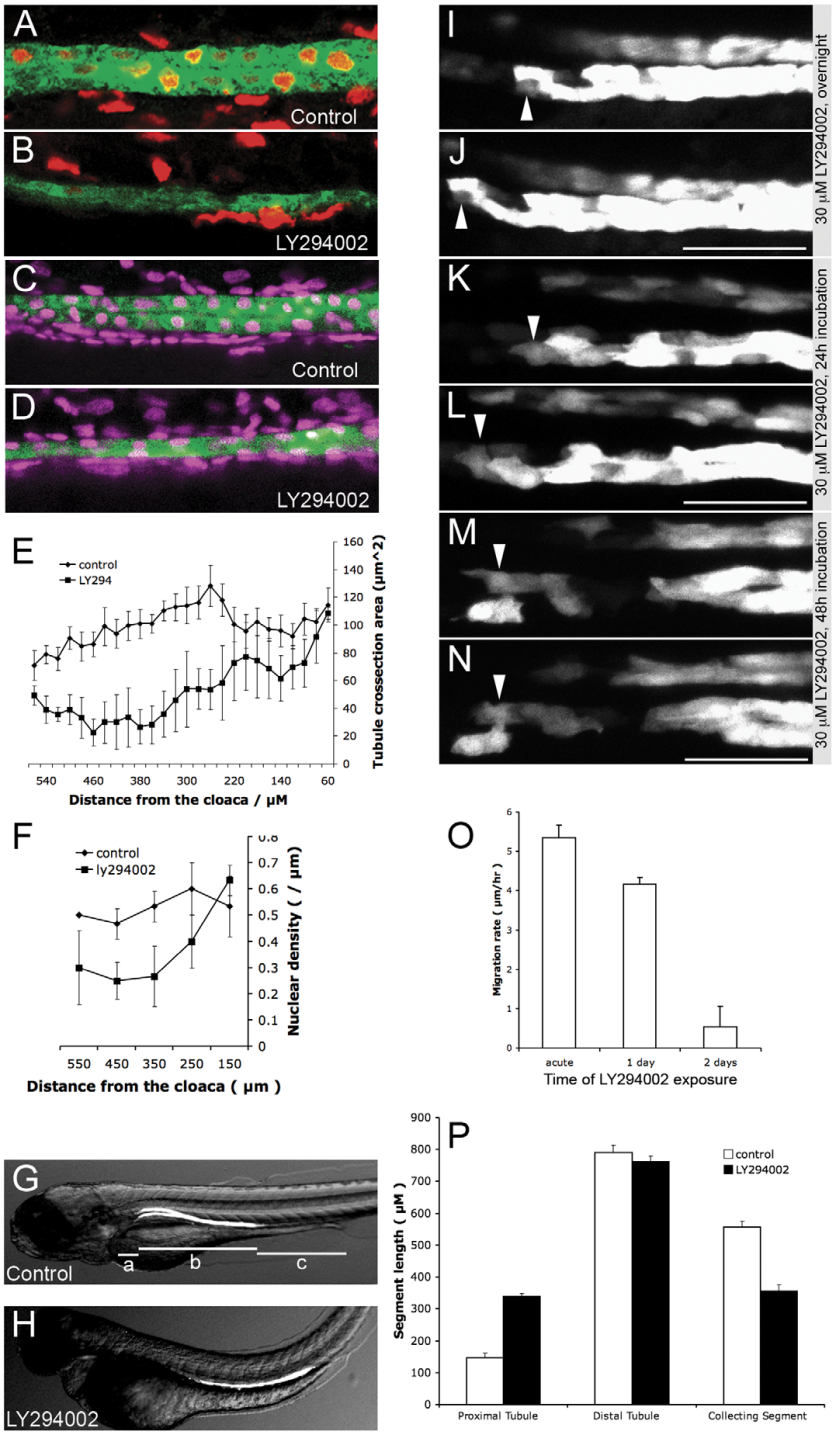
Distal proliferation supports prolonged epithelial migration. (A–D) 1.5 µm confocal slices of the distal tubule. Green: GFP (ET11-9 GFP transgenic in A–B, *Tg(atp1a1a.4:GFP)* transgenic in C&D). Red: BrdU. Magenta: DAPI. (A) BrdU incorporation in the distal tubule (2–3 dpf) in control fish. (B) Lack of BrdU incorporation in distal tubule between (2–3 dpf) when embryo is treated with 30 µM LY294002. Cells outside of the kidney continued to incorporate BrdU. (C) DAPI staining of distal tubule in control fish (4 dpf). (D) DAPI staining of distal tubule treated with 30 µM LY294002 (4 dpf). The distal tubule was markedly thinned in LY294002 condition (B, D). (E) estimated cross-sectional tubule area based on measured maximal diameter of the tubule in confocal stacks at 3 dpf. Circles: control (n = 5). Squares: LY294002 treated fish (n = 3). (F) Linear nuclear density (DAPI) in control fish (circles, n = 3) and LY294002 treated fish (squares, n = 3) confirmed linear stretching of the distal tubule in Ly294002 treated fish (4 dpf). (G, H) Kidney segment lengths in (G) control fish and (H) LY294002 treated fish at 4dpf. ET11-9 transgenic fish were used to estimate the segment lengths: ‘a’, ‘b’ and ‘c’ represent proximal tubule, distal tubule and the pronephric duct. (I–N) Individual frames of time lapse movies of the actively migrating pronephric epithelia in the presence of 30 µM LY294002. Arrowheads point to the individual traced cells. Time lapse immediately after addition of LY294002 (I, J), 24 hours after addition of LY294002 (K, L), and 48 hours after addition of LY294002 (M, N). Frame pairs in I and J, K and L, and M and N are separated by 6 hours. (O) Average epithelial migration rates after different durations of LY294002 exposure. Each bar represents average migration rate of 4 different individual cells in the proximal tubule (ET11-9:GFP). (P) Lengths of proximal tubule (designated ‘a’ in G), distal tubule (designated ‘b’ in G) and pronephric duct (designated ‘c’ in G) in control 96 hpf fish and fish treated with 30 µM LY294002 starting at 30 hpf. White bars: control (n = 9), black bars: LY294002 (n = 9). See also Figure S2.

In prior studies we demonstrated that acute arrest of cell proliferation did not affect kidney epithelial migration [12]. However, the effect of prolonged arrest of cell proliferation in the setting of continued proximal-directed cell migration is unknown. Inhibiting proliferation might be expected to cause stretching or breakdown of the distal nephron epithelium, eventually resulting in distortion of nephron morphology. Prolonged incubation of fish larvae in LY294002 caused a conspicuous thinning of the distal tubule segment (Fig. 3 A–F) while proximal shift of segment boundaries was inhibited (Fig. 3 G, H, P), suggesting that cell migration was restrained by LY294002 treatment. We therefore compared acute and long-term effects of LY294002 treatment on individual cell motility using in vivo time-lapse recordings. We found that the initial rate of pronephric epithelial migration was not significantly affected by LY294002 treatment, indicating that interfering with PI3K signaling did not directly inhibit pronephric cell migration (Fig. 3 I, J, O; movie S3). Instead, the rate of cell migration gradually decreased after 24 h of incubation (Fig. 3 K, L; movie S4) and came to a halt after two days of LY294002 exposure (Fig. 3 M, N; movie S5). Even after collective cell migration was halted, individual cells remained actively protrusive, indicating that LY294002 treatment did not interfere with cytoskeletal dynamics. To confirm the role of cell proliferation in supporting cell migration, we inhibited distal pronephric cell proliferation using the Cdk4/cyclin D1 inhibitor NSC 625987 [20]. This treatment inhibited distal nephron cell proliferation (Fig. S5 B) and generated nephron morphogenesis defects including thinning of the distal tubule and restrained shortening of the proximal nephron, similar to the effects observed when PI3K signaling was inhibited (Fig. S5 A, C). The results strongly suggest that while cell proliferation is not generally required to initiate collective tubule cell migration, cell division in the distal nephron is required to support proximal migration by replacing migrating cells. In the absence of cell proliferation, distal tubule cells, stretched to a maximum length, constrain further collective cell migration. No tubule epithelial cytomegaly was observed with NSC 625987 treatment suggesting that cell division, as opposed to cell growth, is central to the overall tubule volume increase during kidney maturation.

### Modeling Kidney Epithelial Migration and Cell Proliferation

**Figure 4:**
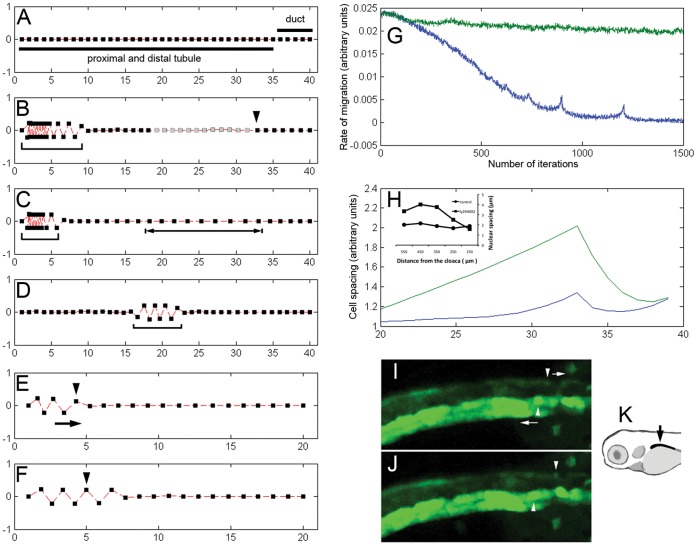
Modeling pronephric migration and proliferation. (A) Initial arrangement of cells in the simulation. Squares: cell positions. All cells in the chain are presumed to be responsive to fluid flow. The difference between the tubule segment (long bar) and the duct segment (short bar) is that cells in the tubule segment migrate faster than cells in the duct segment. (B) Collective migration results in piling up of epithelial cells in the proximal nephron (bracket) and an increase in total number of epithelial cells (gray squares indicate newly formed cells) due to stretch-dependent distal tubule proliferation. The arrowhead points to the zone of proliferation. The number of iterations  = 700. (C) When the threshold for stretch-induced cell proliferation was increased, migration came to a premature halt and, as a result, proximal convolution was significantly reduced (bracket) and distal tubule became overstretched (bracketed arrow). The number of iterations  = 700. (D) When only the distal half of the kidney is subjected to the directional migration bias, the ectopic convolution develops (bracket). The number of iterations  = 100.The starting number of cells in simulations A–D was 40. (E,F) The model predicted that if at any time during active migration (such as in E (100 iterations)) the cue for directional migration (fluid flow) was eliminated, the direction of migration would temporarily reverse (F, arrow in E). (E, F) Arrowheads point to the same cell. The additional number of iterations in F = 100. The starting number of cells in simulations E–F was 20. (G) Simulations predicted that inhibiting distal proliferation should result in premature arrest of migration (lower trace) while in control condition the migration rate remained relatively constant (upper trace). The migration rate was measured over the five cells (20^th–^24^th^ cells counting from the back end of the chain). (H) Inhibiting distal proliferation in live embryos using LY294002 resulted in linear stretch of the distal kidney epithelial cells as evidenced by the increase in the internuclear distance (inset). The same effect is predicted in our simulations (upper trace – no proliferation condition vs. lower trace – control condition). The total number of cells in this simulation  = 40. The distal half is displayed. (I,J) Reversal of the direction of migration can be observed during stochastic transient tubule obstruction. (I) Transiently obstructed tubule (above) and unobstructed tubule (below) in the ET33d10 transgenic fish at time 0. (J) the same two tubules 1 hour later. Arrowheads point to the individual traced cell in each tubule. Arrows indicate the direction of migration. See also Movie S10. (K) Diagram showing the portion of the zebrafish kidney that was imaged in I and J.

The dynamic interaction of nephron fluid flow, collective cell migration, distal tubule stretch, and compensatory cell proliferation led us to develop a computational model linking the forces driving nephron morphogenesis. In this model, inspired by cellular automata, all nephron epithelial cells are made responsive to luminal fluid flow by introducing a migration bias in the direction opposite the direction of flow. In addition, cells in the pronephric tubule (both proximal and distal) are made more migratory compared to cells in the pronephric duct (observed experimentally). Importantly, epithelial cells are presumed to restrict movement of their neighbors by imposing repulsive influence when cell centers get too close (i.e. cell compression) and attractive influence when cell centers are spaced too far apart. Lastly, it is posited that a low-level stochastic cell proliferative activity is signaled by spatial separation (linear stretch) of the cells: when a cell center is distant from the center of its neighbor (stretched), it is more likely to undergo a cell division (Fig. S8, Appendix S1). Based on these principles, the model accurately predicted the behavior of the developing pronephros on a morphogenetic and kinetic level (Fig. 4 A, B; movie S6). As cells begin to migrate, the segment at the interface of actively migrating distal tubule and trailing collecting duct cells generated a ‘hot spot’ of proliferation and a continuous supply of new cells allowed for migration to continue in the proximal direction. When the threshold to this proliferative response was increased, the model accurately predicted the constrained proximal migration observed when PI3K or CDK4/Cyclin D1 signaling was inhibited (Fig. 4 C; movie S7). Unaffected initially, cell migration came to a premature halt when the tubule became maximally stretched (Fig. 4 G, H). Thus, intra–epithelial cell interactions are sufficient to limit collective cell migration in the absence of compensatory cell proliferation and can account for cell spacing and stretch of the distal segment of the collectively migrating tubule (Fig. 3). The model also accurately predicted that a proximal convolution should develop, but would be reduced when cell proliferation is inhibited (compare Fig. 4 C and Fig. 4 B, also Fig. S6).

We showed previously [12] that when proximal luminal fluid flow is eliminated while distant fluid flow remains intact, kidney convolution develops at an ectopic position. Our model predicted this behavior (Fig. 4 D; movie S8). The total stimulus for distal tubule cell proliferation is reduced according to the model (due to decreased length of actively migrating segment and thus, smaller overall distal tubule stretch, supplemental movie S8). This reduction is observed experimentally (Fig. 2 C). Another prediction of the model was that if the stimulus for proximal cell migration (fluid flow) was abruptly removed, tubule cells would respond by re-spacing themselves to equalize cell-cell distance, resulting in reversal of the direction of cell migration (distal-directed migration, Fig. 4 E, F, movie S9). By observing spontaneous transient obstruction events in live imaging of zebrafish transgenics (Fig. 4 I, J, movie S10) and by using distal obstruction to acutely stop fluid flow and remove the stimulus for proximal-directed migration (movie S11), we confirmed this prediction in vivo and observed that the direction of collective cell migration was in fact reversed by acute inhibition of fluid flow.

Lastly, the model also predicted that after removal of Pi3K inhibition, we should expect a burst of cell proliferation and a corresponding burst in migration rates (supplemental Fig. S7 A, B). These predictions were confirmed experimentally (Fig. S7 C). The average peak migration rate after LY294002 removal was greater than 12 µm/hr (Fig. S7 C, movie S12) and occurred 9 hours after LY294002 washout. This peak migration rate, in accordance with model predictions (Fig. S7 B), was much higher than what is observed under normal developmental conditions (6–7 µm/hr). Furthermore, we observed a significant increase in number of cell divisions in distal tubule post- 7 hours after LY294002 washout (movie S13). Using phospho-histone H3 (Ser10) staining to measure the frequency of cell division showed a burst of proliferative activity with a peak at 6.5 hours post-LY294002 removal. Together, these results showed that the effect of Pi3K inhibition on kidney migration and kidney epithelial proliferation is fully reversible. The model did not predict a 6–7 hour delay in the effect of LY294002 washout (Fig. S7 C). This may be due to slow kinetics of LY294002 washout or it may reflect the amount of time required for progression between G1 and M phases after reversal of Pi3K inhibition [21]. In addition, we currently do not have an explanation for a transient reversal of the direction of migration early after LY294002 washout (Fig. S7 C, movie S12). Perhaps, there is another cell population (more proximal) that enters mitosis soon after LY294002 is removed.

## Discussion

Cell migration and proliferation together with cell differentiation are fundamental cellular processes by which tissue and organ morphogenesis occurs. These processes must be tightly coordinated in order for the morphogenesis to proceed properly. Here we presented evidence showing that during kidney morphogenesis and maturation, collective epithelial migration stimulates cell proliferation in the segment adjacent to the rapidly migrating epithelium. This up-regulation of cell proliferation is likely to be signaled by cell stretch and is dependent on PI3K signaling. Cell proliferation is, in turn, required for the full extent of migration to occur. Thus, kidney morphogenesis appears to be a self – regulating process where onset of organ function guides multiple interacting cellular behaviors to result in continued development of organ’s shape. While our data link mechanical stretch to cell proliferation and morphogenesis, we cannot rule out the possibility that additional factors may also play important roles in coupling cell migration and cell proliferation.

The interaction between cell stretch and cell proliferation has been documented in both epithelial and non-epithelial systems [13]. For example, mechanical stretching of epidermal keratinocytes induces cell proliferation [17]. MDCK cells grown as cysts or monolayers show significantly increased cell proliferation when stretched [22]. In addition, endothelial cells proliferate when allowed to spread [23]. There is also evidence pointing to the role for PI3K signaling in linking cell stretch to cell effector responses. For example, AKT is known to be up-regulated in stretched keratinocytes and this up-regulation is dependent on intact PI3K signaling [17]. Here we showed that PI3K signaling is required to link cell migration, cell stretch and cell proliferation in the distal tubule. The precise mechanism by which this linkage of cell stretch and cell proliferation occurs is not known. It has been suggested that PI3K interacts with TrpV4 (a mechanosensitive cation channel) to mediate focal adhesion to focal adhesion signaling [24]. Interestingly, TrpV4 is prominently expressed in the distal kidney [25]. It will be instructive to examine a potential role of TrpV4 in linking cell migration, cell stretch and cell proliferation. PI3K is also known to play a role in mediating cell migration during morphogenesis and repair [26]. To our surprise, we did not observe an acute, direct effect of PI3K inhibition on pronephric epithelial migration. This discrepancy may reflect the fact that pronephric epithelial migration takes place in the absence of leading edge, a common feature of most forms of collective migration [27], [28]. In pronephric migration, every migrating epithelial cell has another cell in front of it. Perhaps, this fundamentally different configuration relies on a different set of signaling factors to organize the migratory behavior. The collective migration model we developed takes into account cell-cell interactions, where both decreased and increased cell spacing profoundly effect cell migratory behaviors. Interestingly, there is recent evidence supporting this concept. It has been shown that there exists cell contact inhibition during collective migration of neural crest cells [29]. In addition, cell culture studies show that changes in cell spacing profoundly affect cell migratory properties [30].

Overall, our findings suggest that the onset of organ function and the resultant mechanical interactions are crucial for organ maturation and continued morphogenesis. Understanding the recursive relationship between organ development and organ function will be critical for uncovering the nature of many developmental disorders and for devising successful approaches to organ engineering and regeneration.

## Materials and Methods

### Zebrafish Care and Transgenic Lines

The *Tg(atp1a1a.4:GFP)* transgenic line was generated as described in [31], the ET(krt8:EGFP)sqet11–9 line and the ET(krt8:EGFP)sqet33-d10 line were a gift from Dr. Vladimir Korzh [15], [16], the ret1:GFP line was a gift from Dr. Shannon Fisher [32], and the *mindbomb (mib^m132^*) mutant line was a gift from Dr. Alan Davidson [31]. All the fish lines were raised and maintained as described in [12], [33]. The *mindbomb*/*Tg(atp1a1a.4:GFP)* line was generated by crossing the corresponding mutant and transgenic lines. The ET(krt8:EGFP)sqet11–9 and ET(krt8:EGFP)-sqet33-d10 lines are referred to as ET11–9 and ET33d10. The embryos for the described experiments were obtained by in-crossing the heterozygous transgenic/mutant fish.

### Chemical and Morpholino Treatment of the Embryos

LY294002 was purchased from Calbiochem, NSC625987 was purchased from Sigma.

LY294002 was diluted to 30 µM and NSC625987 was diluted to 20 µM in E3 water (0.003% PTU), containing 1% DMSO. The chemicals were applied at 30 hpf and the embryos were incubated at 28.5°C. ATG *tnnt2* morpholino (NM_152893): 5′-CATGTTTGCTCTGATCTGACACGCA-3′, was diluted to 0.125 mM, in 100 mM KCl, 10 mM HEPES, 0.1% Phenol Red (Sigma). A fixed volume of 4.6 nl was injected into each embryo at 1–2 cell stage using a Nanoliter2000 microinjector (World Precision Instruments). Morpholino efficacy was confirmed by observing absent heart contractions.

### Modified BrdU Protocol

Decorionated embryos were placed in E3 (0.003%PTU) solution containing 20 µM BrdU and 1% DMSO, buffered to neutral pH with NaHCO3. The embryos were incubated for 24 hours at 28.5°C. The embryos were fixed in Dent’s fixative (80% Methanol, 20% DMSO) overnight. The embryos were then rehydrated into PBS(D)T (1x PBS, 1%DMSO, 0.05% Tween20) and digested with 10 µg/ml of proteinase K for 30 min. The effective proteinase K treatment resulted in embryos adhering to each other. In subsequent manipulations the yolk should fall off the embryo. This does not interfere with imaging or integrity of internal organs. The embryos were washed again and treated with 2N HCl for 30 min. The embryos were washed with PBS(D)T and stained with anti-BrdU antibody.

### Surgical Manipulation of the Embryos

30-hpf embryos were dechorionated and anesthetized with tricaine. An incision just anterior to the cloaca and perpendicular to the long axis of the embryo was made using a razor blade. For anterior obstruction, the incision was made at the level of yolk-to- yolk extension interface. The embryos were allowed to heal for few minutes and then were transferred into new, clean E3 (0.003% PTU) water. We performed anterior obstruction by making an incision perpendicular to the pronephros at the yolk to yolk extension interface in both control and the experimental condition at 1 dpf. The embryos were allowed to develop till 2 dpf at which time BrdU was applied. At 3 dpf the embryos were sorted based on the presence of proximal cysts (positive cysts being indicative of successful obstruction). All the embryos selected for staining had intact truncal circulation to control for possible decreased proliferation due to absent regional blood flow. Distal obstruction was produced by razor blade incision just proximal to the cloaca.

### Antibody Staining

The embryos were blocked with 10% Goat serum (Sigma) overnight at 4°C. After the blocking stage, Goat serum concentration was lowered to 2% and primary antibodies were applied. The antibodies used were: Anti- BrdU mouse monoclonal antibody (clone BU-133, Sigma), anti-GFP rabbit polyclonal antibody (Sigma G1544), anti- phospho histone H3 rabbit monoclonal antibody (clone MC463, Millipore), alpha6F mouse monoclonal antibody (Developmental Studies Hybridoma Bank), and anti-cadherin 17 rabbit polyclonal antibody (Gift from Dr. Zhaoxia Sun, Yale University). All primary antibodies were used at 1∶100 dilution. For confocal imaging, Alexa-labeled secondary antibodies (Invitrogen) were used. All washes were performed using PBS(D)T with 2% goat serum (11).

### Imaging

Imaging was performed using a Zeiss LSM5 Pascal confocal microscope. For time lapse imaging of cell migration, transgenic embryos from in-crossed adults were anesthetized using 0.2 mg/ml tricaine, immobilized/oriented in 2% low melting point agarose, and mounted on the stage of a Zeiss LSM5 Pascal confocal microscope. At 15–30 minute intervals the pronephros was imaged in a z-series stack using a 40X water dipping lens. Maximum intensity projections of each stack were generated and assembled into a time lapse videos using Zeiss pascal software. Frames were additionally processed to adjust contrast and reassembled into quicktime movies using ImageJ (NIH). To image whole mount antibody stained embryos, the washed embryos were placed into PBS(D)T and dehydrated into 100% ethanol. The ethanol was replaced with mounting medium (53% benzyl alcohol (by weight), 45% glycerol (by weight), 2% N-propyl gallate). BrdU localization was studied using 60x oil emersion lens on a confocal microscope.

### Morphometry

The embryos were anesthetized using 0.2 mg/ml Tricaine and immobilized/oriented in 2% low melting point agarose. The embryos were photographed using Leica DFC 300 FX camera mounted on a Leica MZ 16F fluorescent dissecting microscope using Leica Application Suite version 2.4.0 R1 (Leica Microsystems). Images were imported into ImageJ (NIH) and the distance from the posterior edge of the otic vesicle to the proximal edge of the GFP positive pronephric domain was determined for each image. In addition, the length of the GFP positive domain and the distance from the posterior edge of this domain to the cloaca was determined. Measurements were normalized to a stage micrometer measurement. The measurement of the proximal tubule convolution was performed using *Tg(atp1a1a.4:GFP)* transgenic fish. Embryos were anesthetized at 96 hpf, immobilized, and photographed as described above. The images were imported into NIH ImageJ software and curved line lengths were measured using polygon approximation. The index of convolution was estimated as in (12).

The measurement of migration rates was performed using Zeiss Image Examiner software (Carl Zeiss, Inc.). Individual cells were traced in time-lapse images and the distances traveled were measured in relationship to arbitrary stationary reference points in the skin (skin GFP expressing cells). Eight to ten cells were traced per experiment, and the rates of migration were averaged. Morphometric results were plotted using Excel (Microsoft Corp.).

### Modeling

Modeling of cell interactions in the developing pronephros was performed using Matlab software (Mathworks, Inc.). The simulation results were exported into individual frames and reassembled into movies using ImajeJ (NIH).

## Supporting Information

Figure S1 **Proximal progression of the peak of distal tubule proliferation, related to** **Figure 1** **.** BrdU incorporation between 3 and 4 dpf (squares) and between 4 and 5 dpf (circles) shows a shift in the distal tubule proliferation (650 µm vs. 750 µm from the cloaca, arrows).
(TIF)

Figure S2 **Proximal tubule proliferation does not depend on glomerular filtration, related to** **Figure 1** **.** BrdU incorporation was measured in proximal tubule in *tnnt2* morphants between 2.5 and 3.5 dpf squares (n = 7) and compared to that in control fish (rhombi, n = 4). The domain of active proliferation was expanded in morphant fish along the length of the proximal tubule (A). This was most likely due to failed migration in this segment and a subsequent failure of the segment to shorten. The overall amount of BrdU incorporation was not statistically different in *tnnt2* morphants compared to control fish (although the average BrdU incorporation was slightly higher in morphant fish (B, p>0.05). The anterior 125 µm of the proximal tubule was used to count BrdU incorporating cells.
(TIF)

Figure S3 ***mindbomb*** **mutants show robust proliferative response to cell stretch, related to** **Figure 2** **.** Mindbomb mutants were obstructed at 36 hpf and incubated in the presence of BrdU between 48 and 72 hpf. The embryos were then fixed and stained with anti- Cadherin17 (green) and anti- BrdU (red) antibody. Robust BrdU incorporation was observed. Single 1.5 µm confocal slice is shown.
(TIF)

Figure S4 **Obstruction-induced cell stretch does not depend on cell proliferation, related to** **Figure 2** **.** 48 hpf ET33d10 fish were subjected to distal obstruction for 8hours, fixed and stained with anti- pospho histone H3 (green) and anti alpha6F (red) antibodies (A,B). 60 µM Camptothecin was used to inhibit cell proliferation (monitored by the amount of phospho histone H3 staining). Maximal luminal diameter of the proximal kidney was used as a measure of radial cell stretch and was identical in the experimental and the control condition (C). (A,B) show flattened confocal stacks. The apparent kidney staining with phospho histone H3 in (B) is actually in a different focal plane (in the CNS). No significant kidney phospho histone H3 staining is observed after 8hours of obstruction.
(TIF)

Figure S5 **Inhibiting CDK4/CyclinD1 signaling phenocopies the effects of inhibiting PI3K, related to** **figure 3** **.** Inhibiting CDK4/CyclinD1 using 20 µM NSC625987 results in exaggerated linear stretching of the distal tubule (A), ET11-9 transgenic. This chemical treatment also resulted in significant reduction in distal kidney BrdU incorporation (B), P<0.05. (C): the segment lengths were affected by NSC625987 treatment in a manner very similar to LY294002. The proximal tubule becomes significantly longer (P<0.01), the collecting duct becomes significantly shorter (P<0.01). Unlike the LY294002 result, the distal tubule is slightly shorter in NSC625987 treatment condition. This small, but statistically significant effect (P<0.05) could be due to small direct effect of NSC625987 on the migration rate (distal tubule elongation is migration dependent).
(TIF)

Figure S6 **Partial inhibition of proximal convolution by LY294002, related to** **figure 4** **.** *Tg(atp1a1a.4:GFP)* transgenics were analysed for the presence and the extent of proximal convolution at 4 dpf in control embryos (A) vs. the LY294002 treated embryos (B). The estimation of the degree of convolution by a convolution index shows partial but significant reduction in the degree of proximal convolution (C, P<0.01).
(TIF)

Figure S7 **Rebound burst in cell proliferation and migration after LY294002 removal, related to** **figures 2** **–** **4** **.** Computational modeling predicted a burst in cell proliferation (A) and a corresponding burst in cell migration (B) after the reversal of the suppression of proliferation. Arrowheads point to when the inhibition of proliferation is removed. (C, circles): Removal of LY294002 resulted in burst of cell proliferation between 6 and 7 hours after the washout. 30 µM LY294002 was applied at 30 hpf and removed at 3 dpf. Distal-most 700 µm of kidney epithelia were evaluated using alpha6F/phospho histone H3 antibody stains. The number of positive nuclei was counted at 2 hr (n = 4), 3.5 hr (n = 2), 5 hr (n = 6), 6.5 hr (n = 3), 9 hr (n = 3) and 11.5 hr (n = 3) and compared to control (3.75+/−0.98, n = 4) and 30 µM LY294002 condition (2.33+/−1.08, n = 3). A ‘spike’ of cell proliferation is observed between 6 and 7 hours after LY294002 removal. After that proliferation returns to control levels. A residual proliferation in the presence of LY294002 is likely due to the inclusion of pronephric duct in the overall counts. (C, squares): Average migration rates after LY294002 washout (n = 4 cells).
(TIF)

Figure S8 **Basic assumptions of the computational model, related to** **figure 4** **.** Each cell (cell centers are represented by small dark circles, A–D) can randomly move, assuming (after each iteration) a new position anywhere within an area (determined by cell agility) centered on the cell itself (unbiased scenario, A). Fluid flow introduces a bias, such that the center of the area representing potential new positions of a cell is shifted with regard to its center (B). Similarly, when a cell is positioned at a ‘neutral’ distance from its neighbors ‘A’ and ‘B’, it can move in an unbiased fashion (C). However, when it finds itself too close to its neighbor (‘A’ in D) or too far from its neighbor (‘B’ in D), it will be biased to move away from or towards a corresponding cell.
(TIF)

Movie S1 **Time lapse movie of the distal-most tubule of the zebrafish pronephros between 2.5 and 3 dpf, related to** **figure 1** **.** The double-head arrow shows stretching of this segment of the epithelium due to the difference in migration rates of two sides of this domain. The transgenic line used was *Tg(atp1a1a.4:GFP)*. The head is to the left. Frame interval is 30 min. Number of frames is 24. Each frame is a flattened confocal stack.
(MOV)

Movie S2 **Time lapse movie of the pronephric tubule in** ***mindbimb*** **homozygous mutant fish between 2.5 and 3 dpf, related to** **figure 2** **.** *Tg(atp1a1a.4:GFP)* was used to track the kidney epithelium. The apparent gap in the epithelium is due to expansion of multiciliated cells which are not fuorescent. The directed migration if essentially stopped in this mutant fish. The head is to the left. Frame interval is 30 min. Number of frames is 29. Each frame is a flattened confocal stack.
(MOV)

Movie S3 **Time lapse movie of ET11-9 transgenic fish between 2.5 and 3** **dpf immediately after application of 30 µM LY294002, related to** **figure 3** **.** The head is to the left. Frame interval is 20 min. Number of frames is 42. Each frame is a flattened confocal stack.
(MOV)

Movie S4 **Time lapse movie of ET11-9 transgenic fish between 2.5 and 3** **dpf, 24 hours after application of 30 µM LY294002, related to** **figure 3** **.** The head is to the left. Frame interval is 20 min. Number of frames is 28. Each frame is a flattened confocal stack.
(MOV)

Movie S5 **Time lapse movie of ET11-9 transgenic fish between 3.5 and 4** **dpf, 48 hours after application of 30 µM LY294002, related to** **figure 3** **.** The head is to the left. Frame interval is 20 min. Number of frames is 28. Each frame is a flattened confocal stack.
(MOV)

Movie S6 **Simulation of the kidney epithelial migration using computational model described in Appendix S1, related to** **figure 4** **A, B.** A default parameter set is used here (Appendix S1). Ten iterations of the algorithm were used to produce each sequential frame. Number of frames is 101.
(MOV)

Movie S7 **Simulation of the kidney epithelial migration with decreased proliferation using computational model described in Appendix S1, related to** **figure 4** **C.** The value of the “threshold” parameter was increased from 4.5 (default) to 6.5. Ten iterations of the algorithm were used to produce each sequential frame. Number of frames is 101.
(MOV)

Movie S8 **Simulations predict the development of ectopic convolution when proximal fluid flow is absent, related to** **figure 4** **D.** The “skew” factor is non-zero in the second (right) half of the chain and zero in the first (left) half. The ectopic convolution develops at the interface of the migrating and non-migrating groups. Ten iterations of the algorithm were used to produce each sequential frame. Number of frames is 21.
(MOV)

Movie S9 **Simulation of the kidney epithelial migration when luminal flow is temporarily stopped, related to** **figure 4** **E, F.** The value of “skew” factor was changed from 0.1 to 0 after 100^th^ iteration (frame 10) and back to 0.1 after 300^th^ iteration (frame 30), leading to transient reversal of the direction of migration between frames 11 and 30. Ten iterations of the algorithm were used to produce each sequential frame. Number of frames is 42.
(MOV)

Movie S10 **Time lapse movie of ET33d10 transgenic fish between 3.5 and 4** **dpf, related to** **figure 4** **I, J**. One of the proximal tubules is transiently obstructed for part of the time lapse and during this time the direction of migration is reversed. The migration returns to normal after this transient obstruction is resolved. The head is to the left. Frame interval is 20 min. Number of frames is 28. Each frame is a flattened confocal stack.
(MOV)

Movie S11 **Time lapse movie of ET11-9 transgenic fish between 1.5 and 2** **dpf, immediately after mechanically obstructing the kidney, related to** **figure 4** **.** Transient backward migration of the obstructed epithelium is seen. The head is to the left. Frame interval is 20 min. Number of frames is 33. Each frame is a flattened confocal stack.
(MOV)

Movie S12 **Time lapse movie of ET11-9 transgenic fish between 3 and 3.5** **dpf, immediately after removal of LY294002.** 30 µM LY294002 was applied at 30 hpf and removed at 3 dpf. The head is to the left. Frame interval is 20 min. Number of frames is 43. Each frame is a flattened confocal stack.
(MOV)

Movie S13 **Time lapse movie of ET11-9/8 kb transgenic fish (distal tubule) after LY294002 removal.** 30 µM LY294002 was applied at 30 hpf and removed at 2.5 dpf. The recording was started 6hours after removal of LY294002. Flashing arrows point to dividing cells. The head is to the left. Frame interval is 20 min. Number of frames is 42. Each frame is a flattened confocal stack.
(MOV)

Appendix S1 **Model of kidney epithelial migration.**
(DOCX)
