# Supplementary figures and images for: Mechanical Stretch and PI3K Signaling Link Cell Migration and Proliferation to Coordinate Epithelial Tubule Morphogenesis in the Zebrafish Pronephros
Source: PLoS One. 2012 Jul 18;7(7):e39992. doi: 10.1371/journal.pone.0039992 (PMC3399848; doi:10.1371/journal.pone.0039992)

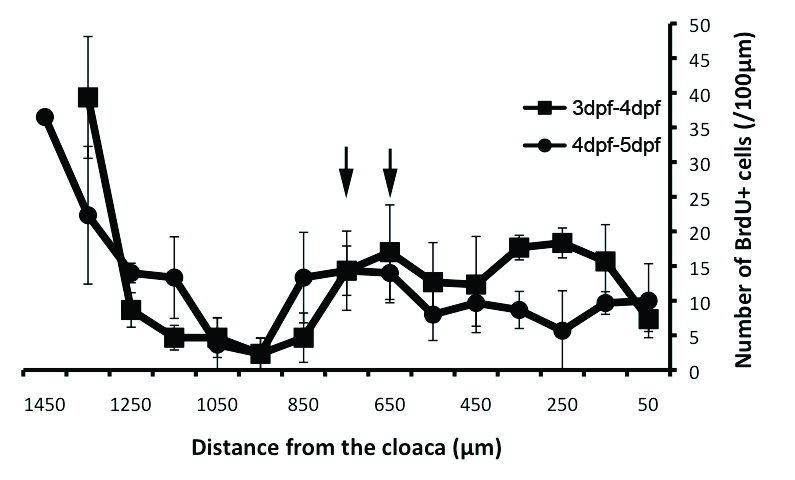

Supplement: Figure S1 — Proximal progression of the peak of distal tubule proliferation, related to Figure 1 . BrdU incorporation between 3 and 4 dpf (squares) and between 4 and 5 dpf (circles) shows a shift in the distal tubule proliferation (650 µm vs. 750 µm from the cloaca, arrows). (TIF) [file pone.0039992.s001.tif]

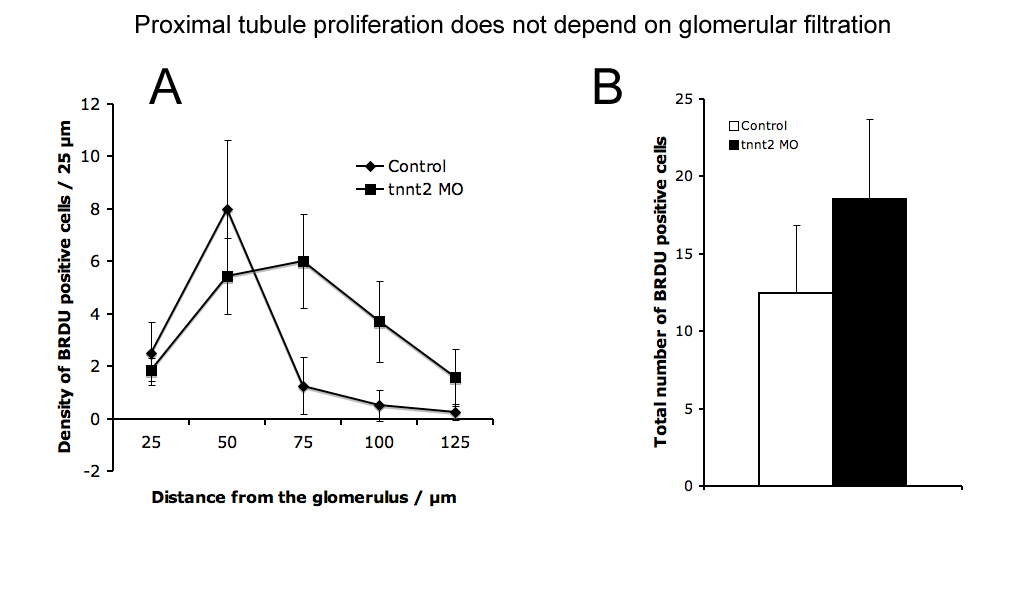

Supplement: Figure S2 — Proximal tubule proliferation does not depend on glomerular filtration, related to Figure 1 . BrdU incorporation was measured in proximal tubule in tnnt2 morphants between 2.5 and 3.5 dpf squares (n = 7) and compared to that in control fish (rhombi, n = 4). The domain of active proliferation was expanded in morphant fish along the length of the proximal tubule (A). This was most likely due to failed migration in this segment and a subsequent failure of the segment to shorten. The overall amount of BrdU incorporation was not statistically different in tnnt2 morphants compared to control fish (although the average BrdU incorporation was slightly higher in morphant fish (B, p>0.05). The anterior 125 µm of the proximal tubule was used to count BrdU incorporating cells. (TIF) [file pone.0039992.s002.tif]

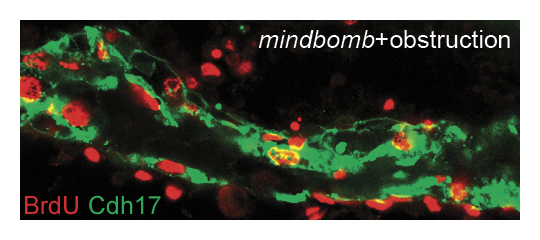

Supplement: Figure S3 — mindbomb mutants show robust proliferative response to cell stretch, related to Figure 2 . Mindbomb mutants were obstructed at 36 hpf and incubated in the presence of BrdU between 48 and 72 hpf. The embryos were then fixed and stained with anti- Cadherin17 (green) and anti- BrdU (red) antibody. Robust BrdU incorporation was observed. Single 1.5 µm confocal slice is shown. (TIF) [file pone.0039992.s003.tif]

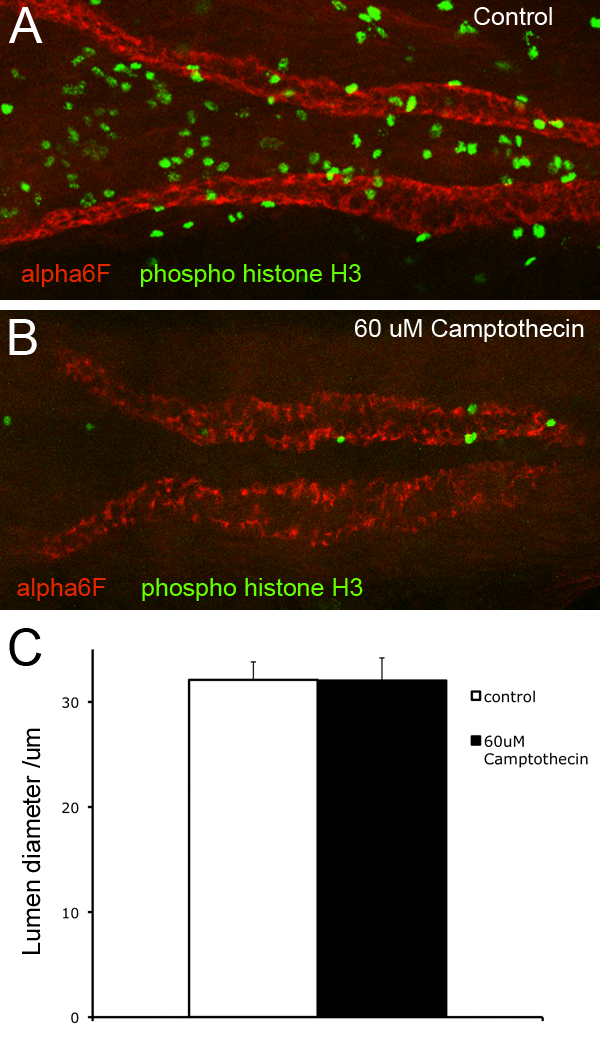

Supplement: Figure S4 — Obstruction-induced cell stretch does not depend on cell proliferation, related to Figure 2 . 48 hpf ET33d10 fish were subjected to distal obstruction for 8hours, fixed and stained with anti- pospho histone H3 (green) and anti alpha6F (red) antibodies (A,B). 60 µM Camptothecin was used to inhibit cell proliferation (monitored by the amount of phospho histone H3 staining). Maximal luminal diameter of the proximal kidney was used as a measure of radial cell stretch and was identical in the experimental and the control condition (C). (A,B) show flattened confocal stacks. The apparent kidney staining with phospho histone H3 in (B) is actually in a different focal plane (in the CNS). No significant kidney phospho histone H3 staining is observed after 8hours of obstruction. (TIF) [file pone.0039992.s004.tif]

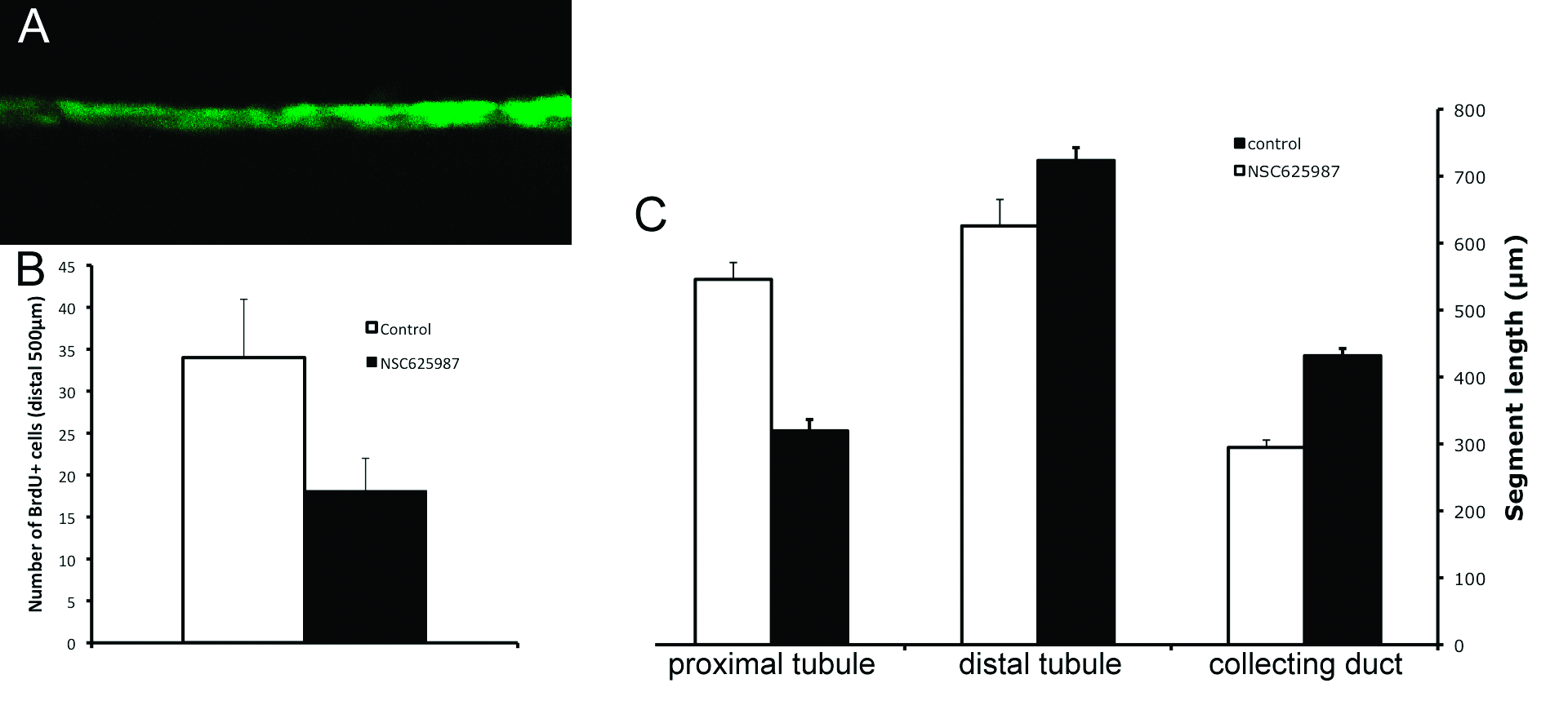

Supplement: Figure S5 — Inhibiting CDK4/CyclinD1 signaling phenocopies the effects of inhibiting PI3K, related to figure 3 . Inhibiting CDK4/CyclinD1 using 20 µM NSC625987 results in exaggerated linear stretching of the distal tubule (A), ET11-9 transgenic. This chemical treatment also resulted in significant reduction in distal kidney BrdU incorporation (B), P<0.05. (C): the segment lengths were affected by NSC625987 treatment in a manner very similar to LY294002. The proximal tubule becomes significantly longer (P<0.01), the collecting duct becomes significantly shorter (P<0.01). Unlike the LY294002 result, the distal tubule is slightly shorter in NSC625987 treatment condition. This small, but statistically significant effect (P<0.05) could be due to small direct effect of NSC625987 on the migration rate (distal tubule elongation is migration dependent). (TIF) [file pone.0039992.s005.tif]

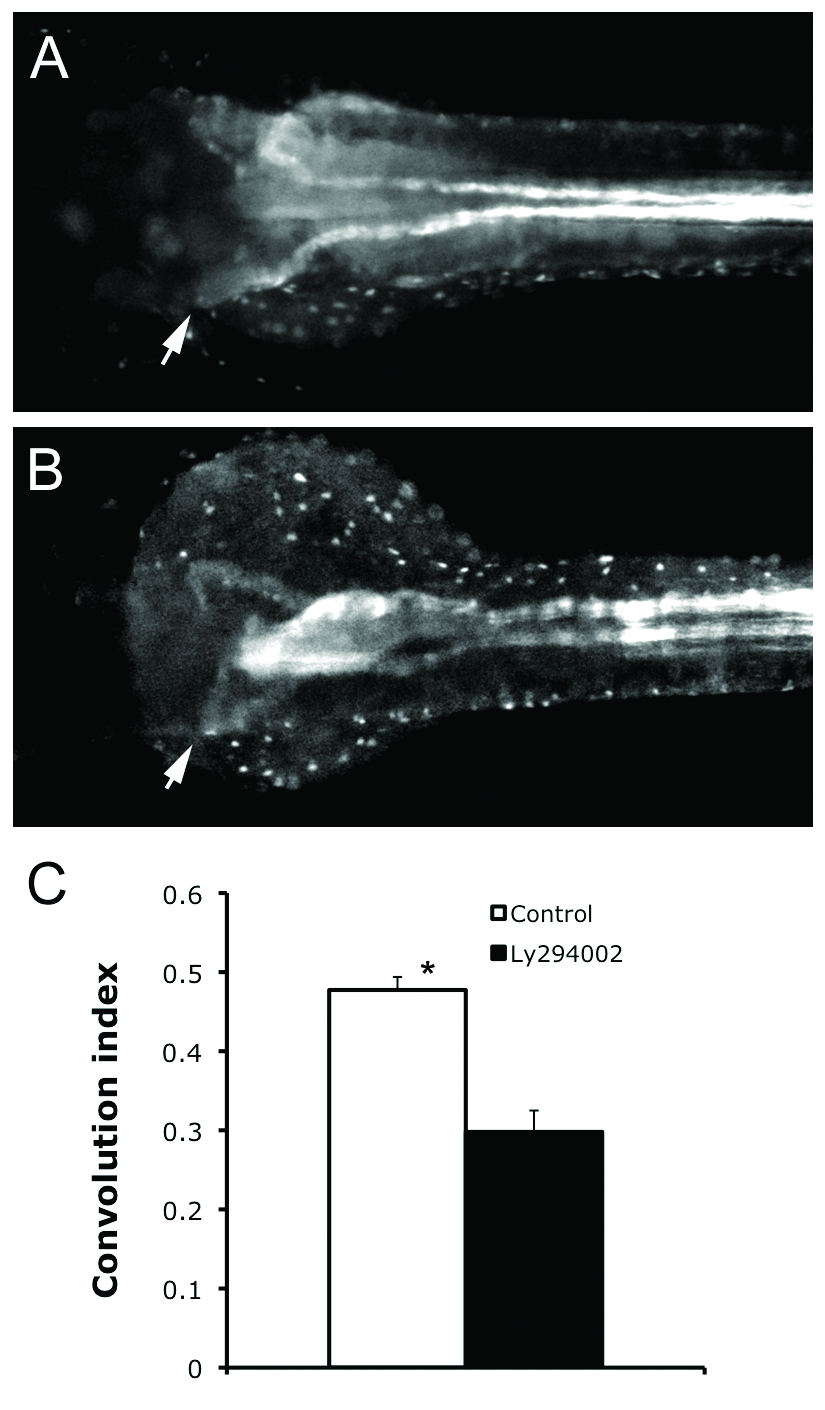

Supplement: Figure S6 — Partial inhibition of proximal convolution by LY294002, related to figure 4 . Tg(atp1a1a.4:GFP) transgenics were analysed for the presence and the extent of proximal convolution at 4 dpf in control embryos (A) vs. the LY294002 treated embryos (B). The estimation of the degree of convolution by a convolution index shows partial but significant reduction in the degree of proximal convolution (C, P<0.01). (TIF) [file pone.0039992.s006.tif]

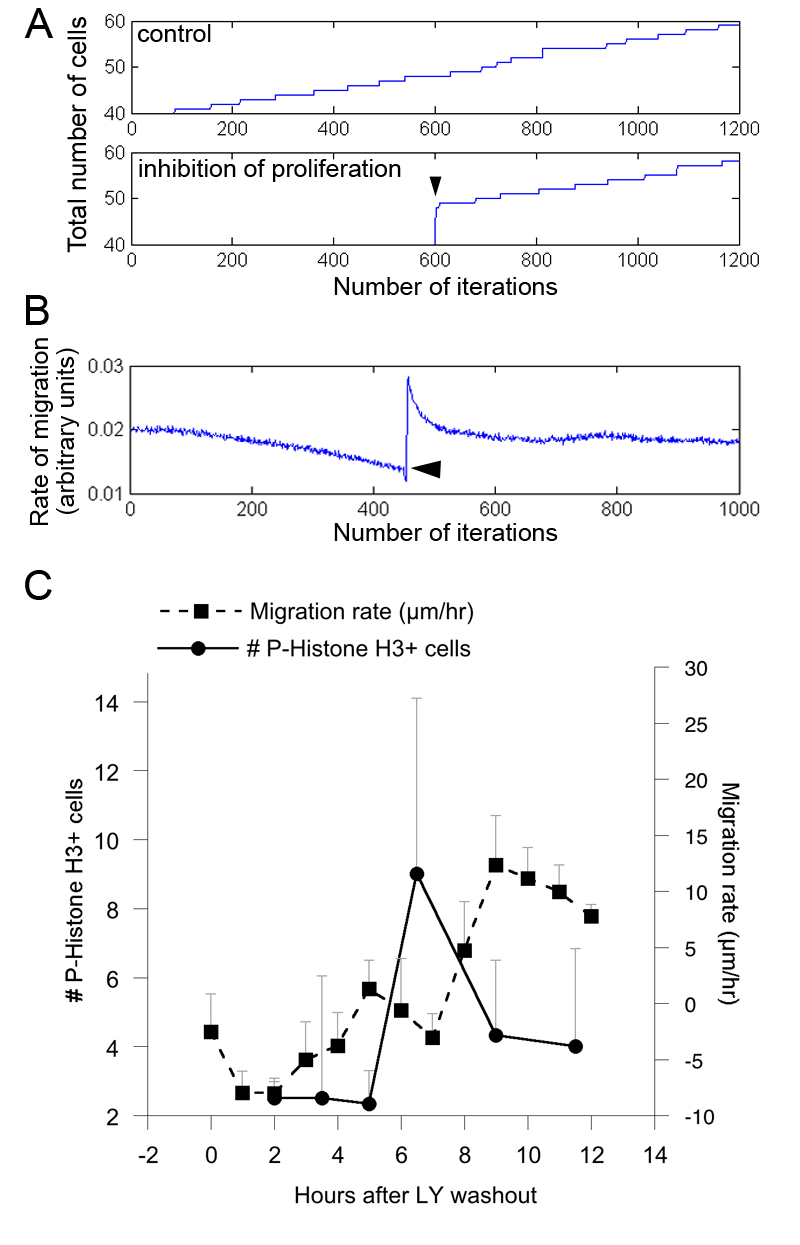

Supplement: Figure S7 — Rebound burst in cell proliferation and migration after LY294002 removal, related to figures 2 – 4 . Computational modeling predicted a burst in cell proliferation (A) and a corresponding burst in cell migration (B) after the reversal of the suppression of proliferation. Arrowheads point to when the inhibition of proliferation is removed. (C, circles): Removal of LY294002 resulted in burst of cell proliferation between 6 and 7 hours after the washout. 30 µM LY294002 was applied at 30 hpf and removed at 3 dpf. Distal-most 700 µm of kidney epithelia were evaluated using alpha6F/phospho histone H3 antibody stains. The number of positive nuclei was counted at 2 hr (n = 4), 3.5 hr (n = 2), 5 hr (n = 6), 6.5 hr (n = 3), 9 hr (n = 3) and 11.5 hr (n = 3) and compared to control (3.75+/−0.98, n = 4) and 30 µM LY294002 condition (2.33+/−1.08, n = 3). A ‘spike’ of cell proliferation is observed between 6 and 7 hours after LY294002 removal. After that proliferation returns to control levels. A residual proliferation in the presence of LY294002 is likely due to the inclusion of pronephric duct in the overall counts. (C, squares): Average migration rates after LY294002 washout (n = 4 cells). (TIF) [file pone.0039992.s007.tif]

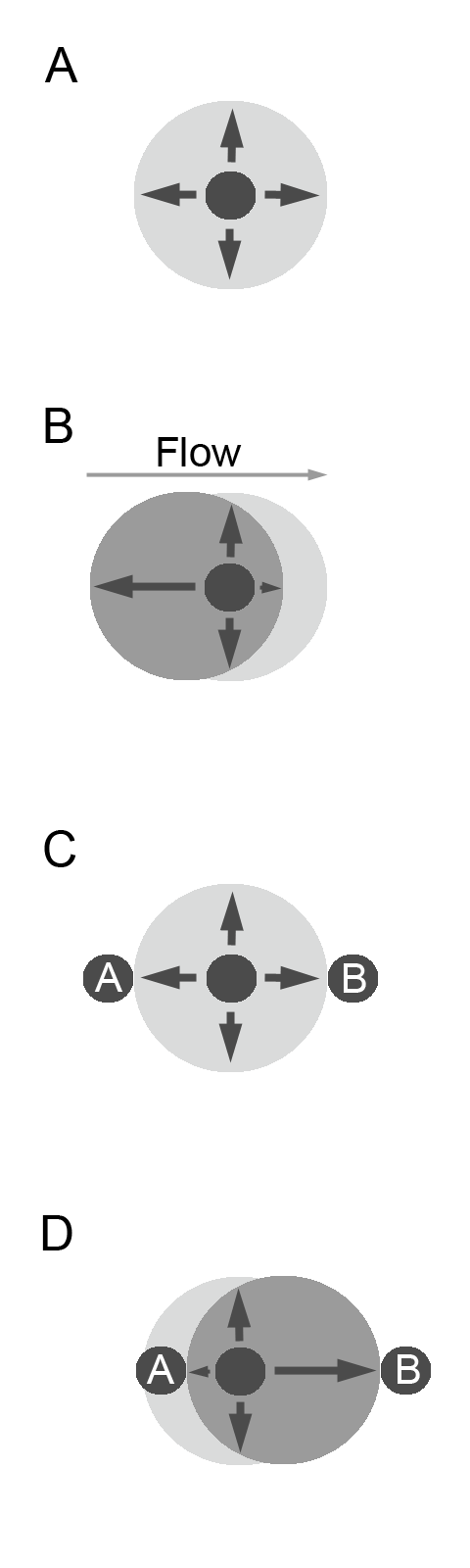

Supplement: Figure S8 — Basic assumptions of the computational model, related to figure 4 . Each cell (cell centers are represented by small dark circles, A–D) can randomly move, assuming (after each iteration) a new position anywhere within an area (determined by cell agility) centered on the cell itself (unbiased scenario, A). Fluid flow introduces a bias, such that the center of the area representing potential new positions of a cell is shifted with regard to its center (B). Similarly, when a cell is positioned at a ‘neutral’ distance from its neighbors ‘A’ and ‘B’, it can move in an unbiased fashion (C). However, when it finds itself too close to its neighbor (‘A’ in D) or too far from its neighbor (‘B’ in D), it will be biased to move away from or towards a corresponding cell. (TIF) [file pone.0039992.s008.tif]
