# Supplementary material for: Mechanical Stretch and PI3K Signaling Link Cell Migration and Proliferation to Coordinate Epithelial Tubule Morphogenesis in the Zebrafish Pronephros
Source: PLoS One. 2012 Jul 18;7(7):e39992. doi: 10.1371/journal.pone.0039992 (PMC3399848; doi:10.1371/journal.pone.0039992)
Supplement: Appendix S1 — Model of kidney epithelial migration. (DOCX) [file pone.0039992.s022.docx]

**Model of kidney epithelial migration:**

This simple model assumes that cells can migrate within two-dimensional space. Each cell shifts its location randomly around the cell’s current position (Fig S7 A). The value of r reflects cell ‘agility’. The ‘unbiased’ displacement is calculated as:

Δx=random(-r/r), Δy=random(-r/r)

(random(-r/r) is a random real number between -r and r).

The fluid flow (X axis) introduces directional bias to this displacement, represented by a darker circle (Fig S7, B). Thus the cell is more likely to move left (opposite the direction of the flow, long arrow):

Δx’=Δx+skew, Δy’=Δy

Finally, cell movement is presumed to be influenced by its proximity to neighboring cells (Fig S7 C,D). When a cell is found at a ‘neutral’ distance from the adjacent cells, migration of this cell is not affected by the neighbors (Fig S7, C). However, when a cell gets too close to one of its neighbors, it becomes biased to move away from it. Alternatively, when a cell gets too far away from one of its neighbors, it becomes biased to move towards it. We assumed a linear response of cells to stretch/crowding. If the distance between the two cells is R, the ‘neutral’ spacing is N and dR=N-R, then:

Δx’’=Δx’+sf*dR*R_x_/R, Δy’’=Δy’+sf*dR*R_y_/R

(R_x_ and R_y_ are the X and the Y components of R and sf (‘stretch factor’) determines how sensitive the cell is to being compressed or stretched). The value of sf is kept the same for cell stretching and cell compression. This is done for purposes of simplicity.

Finally, we postulated that a low level baseline proliferation is signaled by cell stretch. If the distance between a cell and the two of its neighbors is R1 and R2, and the baseline proliferation is governed by a proliferation index Pi, we can define value:

S=R1+R2+random(0/Pi)

If S becomes larger than a given threshold value, the cell divides into two. The threshold value is dependent on external factors such as presence of inhibitors of cell proliferation. The proliferation index Pi is intrinsic to a given cell population and can in principal change from segment to segment. In our simulations we posed a uniform low value for Pi such that in the absence of cell stretch no cell ever reaches a threshold to divide. This was done to simplify the picture and model the proliferative behavior of distal tubule specifically.

We used these simple assumptions to model the behavior of pronephric epithelium. The full code that was used to implement the model is shown below.

It is broken into two scripts used to produce the simulations in figure 4. Each script has a header (File1 and File2). The body of the text following each header can be copy-pasted into a matlab text editor and executed within a matlab environment. The first script should be run once and the second can be run repeatedly until the desired state of the system is achieved. The actual code is in bold.

**%File1:**

%This script takes care of a few global variables and sets up

%the initial "cell" arrangement

%First, we delete all the variables and clear the program memory:

**clear();**

%Then we define the total number of cells in the simulation (xmax) and %the length of the chain (xlength) and set the

%iteration counter (t) to 0:

**xmax=40;**

**xlength=xmax;**

**t=0;**

%We also define how many of these cells are duct cells,

%which are slower migrating

**nduct=6;**

%We set the baseline proliferation index (pi) that defines the

%likelihood of spontaneous cell division (when combined with a %threshold value, which is defined within the second script):

**pi=2;**

%We also define the "normal" distance between cells.

%The cells will react to deviations from this "ideal" distance

**normal_spacing=1;**

%This factor imposes tissue constrains on lateral travel

%(for example, the value of 0 would only allow cell to move within

%a straight line:

**ylimit=0.2;**

%This factor sets a limit on longitudinal compressability of the %chain. Without it cells can pass slip each other, braking down the %integrity of the chain.

**xlimit=0.2;**

%The “n_iter” factor defines the number of iterations before the

%second script stops and displays the outcome.

**n_iter=10;**

%"Stretch" factor (sf) defines how vigorously a cell will react to

%being too close or too far away from it's neighbor

**sf=1;**

%Finally, we set the starting conditions, putting all the "cells" in a

%straight line, all spaced by 1 (we do not use any explicit units in

%this simulation, however, they can be easily added. The xd(i), yd(i) %define an XY position of a given cell, while xd1(i) and yd1(i) define

%it's next XY position.

**for i=1:1:xmax**

**xd(i)=i;**

**xd1(i)=i;**

**yd(i)=0;**

**yd1(i)=0;**

**end**

**%File2:**

%The global control parameter "skew" defines directional bias

%secondary to fluid flow (skew). The "start_flow" parameter

%defines where along the length of the tubule fluid flow begins.

**skew=0.1;**

**start_flow=1;**

%The "threshold" parameter determines the likelihood of a given cell

%to divide (below).

**threshold=4.5;**

**for count=1:1:n_iter**

%This will count the total number of iterations (t):

**t=t+1;**

%For each "cell" we generate two random numbers (between 0 and 0.01).

%These will define a random component of cell migration.

**r=rand(xmax,2)/100-0.005;**

%"Decision" algorithm for each cell in the migrating group:

%The cycle is between "cell" #2 and "cell" #(nmax-1) because

%the first and the last "cells" in the group are fixed in space

**for i=2:1:(xmax-1)**

%First we decide if we are looking at tubule vs. duct cells because

%duct cells migrate slower (defined by factor "a". a=0.2 for tubule

%and 0.01 for the duct:

**if (i>1)&&(i<xmax-nduct)**

**a=0.2;**

**else**

**a=0.01;**

**end**

%Here we define what segment of the kidney "senses" fluid flow:

**if i>start_flow**

**r(i,1)=r(i,1)-skew;**

**end**

%Below we define how relative position of the adjacent cells

%influences cell movement.

%First we define the interactions at the front of a given cell.

%Distance (x axis) between a cell (i) and the one in front of it

%(i-1):

**xfront(i)= xd(i)-xd(i-1);**

%Distance (y axis) between a cell (i) and the one in front of it

%(i-1):

**yfront(i)= yd(i)-yd(i-1);**

%Total distance between the cell and the one in front of it (front(i):

**front(i)=sqrt((xfront(i))^2+(yfront(i))^2);**

%Deviation (dfront(i)) between the inter-cell distance and the normal

%spacing (normal_spacing):

**dfront(i)=normal_spacing-front(i);**

%X and Y components of the stretch/crowding response

%The cells that descended from one cell (and thus have an identical

%location are first separated by a small amount to prevent

%unpredictable behaviors:

**if front(i)==0**

**dxfront(i)=0.1;**

**dyfront(i)=0;**

%All the other cells:

**else**

**dxfront(i)=sf*dfront(i)*xfront(i)/(front(i));**

**dyfront(i)=sf*dfront(i)*yfront(i)/(front(i));**

**end**

%Second, we define the interactions at the back of a given cell.

%Distance (x axis) between a cell (i) and the one behind it (i+1):

**xback(i)=xd1(i+1)-xd1(i);**

%Distance (y axis) between a cell (i) and the one behind it (i+1):

**yback(i)=yd1(i+1)-yd1(i);**

%The totat distance:

**back(i)=sqrt((xback(i))^2+(yback(i))^2);**

%Deviation (dback(i)) between the inter-cell distance and the normal

%spacing (normal_spacing):

**dback(i)= normal_spacing-back(i);**

%This describes the behavior of just divided cells:

**if back(i)==0**

**dxback(i)=-0.1;**

**dyback(i)=0;**

%All the other cells:

**else**

**dxback(i)=sf*dback(i)*xback(i)/(back(i));**

**dyback(i)=sf*dback(i)*yback(i)/(back(i));**

**end**

%The following two lines combine the influence of the front and the

%back neighbor cells (X and Y) axes:

**dxp(i)=-dxback(i)+dxfront(i);**

**dyp(i)=-dyback(i)+dyfront(i);**

%Finally, the change in cell positions:

**rx(i)=(r(i,1) +dxp(i))*a;**

**ry(i)=((r(i,2))+dyp(i))*a;**

%Limit checks to prevent 'tissue disintegration':

**if xfront(i)<xlimit**

**rx(i)=0;**

**end**

**if (abs(yd(i))>ylimit)**

**ry(i)=0;**

**end**

%The new positions:

**xd1(i)=xd(i)+rx(i);**

**yd1(i)=yd(i)+ry(i);**

%The longitudinal rate of migration (since it is defined per iteration

%cycle can be calculated as:

**xspeed(i)=xd(i)-xd1(i);**

**end**

% Cell proliferation module. For each cell a decision will be made to

%divide on not based on it's separation from adjacent cells

**ct=xmax;**

**for k=2:1:(ct-1)**

**str(k)=xd1((k+1))-xd1((k-1))+rand()*pi;**

**if(str(k)>threshold)**

**ct=ct+1;**

**for l=(ct):-1:(k+1)**

**xd1(l)=xd1((l-1));**

**yd1(l)=yd1((l-1));**

**end**

**end**

**end**

%After we completed the cycle of migration/division, we assign the new

%xy values to each cell:

**xd=xd1;**

**yd=yd1;**

%We also update the total number of cells:

**xmax=ct;**

%Lastly, we compute the everage speed over a range of cell numbers

%(cells 20-24 counting from the back, this works well for xmax=40):

**v(t)=mean(xspeed(xmax-24:xmax-20));**

**end**

%The inter-cell distance:

**xdist=diff(xd);**

%Plotting module:

%This panel plots cell positions after each (n_iter) iteration cycles:

**subplot(3,1,1),**

**im=plot(xd(:), yd(:), '--rs','LineWidth',1,'MarkerEdgeColor','k','MarkerFaceColor','k','MarkerSize',4);**

**axis([0 xlength+1 -1 1]);**

%This panel plots inter-cell distances along the x axis:

**subplot(3,1,2),**

**check1=size(xdist);**

**if check1(2)==(xmax-1)**

**plot(xdist);**

**end**

%This last panel plots the average rates of migration in a given

%segment:

**subplot(3,1,3),**

**time=size(v);**

**x=1:1:time;**

**plot(1:time(2),v);**
